# Development of Highly pH-Sensitive Hybrid Membranes by Simultaneous Electrospinning of Amphiphilic Nanofibers Reinforced with Graphene Oxide

**DOI:** 10.3390/jfb10020023

**Published:** 2019-05-21

**Authors:** Mohsen Gorji, Ali Sadeghianmaryan, Hossein Rajabinejad, Saman Nasherolahkam, Xiongbiao Chen

**Affiliations:** 1 New Technologies Research Center (NTRC), Amirkabir University of Technology, Tehran 15914, Iran; mgorji80@yahoo.com (M.G.); Saman.nasher@gmail.com (S.N.); 2 Department of Chemistry, Ardabil Branch, Islamic Azad University, Ardabil 5615731567, Iran; 3 The Centre for Research on Adaptive Nanostructures and Nanodevices (CRANN), Trinity College Dublin, University of Dublin, Dublin 2, Ireland; hoseinrnejad@gmail.com; 4 Department of Mechanical Engineering, University of Saskatchewan, Saskatoon, SK S7N5A9, Canada

**Keywords:** pH sensitive, protective clothing, wound dressing, bimodal membrane

## Abstract

Nanofibrous-based pH sensors have shown promise in a wide range of industrial and medical applications due to their fast response time and good mechanical properties. In the present study, we fabricated pH-sensitive sensors of nanofibrous membranes by electrospinning polyurethane (PU)/poly 2-acrylamido-2-methylpropanesulfonic acid (PAMPS)/graphene oxide (GO) with indicator dyes. The morphology of the electrospun nanofibers was examined using scanning electron microscopy (SEM). The effect of hydrophilic polymer ratio and concentration of GO on the sensing response time was investigated. The sensitivity of the membranes was studied over a wide pH range (1–8) in solution tests, with color change measured by calculating total color difference using UV-vis spectroscopy. The membranes were also subjected to vapor tests at three different pH values (1, 4, 8). SEM results show the successful fabrication of bimodal fiber diameter distributions of PU (mean fiber diameter 519 nm) and PAMPS (mean fiber diameter 78 nm). Sensing response time decreased dramatically with increasing concentrations of PAMPS and GO. The hybrid hydrophobic/hydrophilic/GO nanofibrous membranes are capable of instantly responding to changes in solution pH as well as detecting pH changes in chemical vapor solution in as little as 7 s.

## 1. Introduction

Electrospun nanofibrous membranes (ENMs) are an important category of fibrous structures that can be produced via electrospinning. Due to their ease of production, interconnected porosities, and high surface area, ENMs have found a wide range of applications. Some of these applications, most notably protective clothing (PC) and wound dressing, require smart ENMs with sensing properties, such as detecting pH changes in the environment. Using pH-sensitive nanofibrous membranes in the PC sector helps the wearer be more aware of chemical pollution in the surrounding atmosphere, leading to better protection. This function can be very useful in clothing designed to protect against chemical agents and industrial chemical hazards. In the wound treatment sector, creating dressings with an indicator function that can sense the status of the skin and convey the information to the healthcare professional can reduce the frequency of dressing changes [1].

Studies have been carried out to fabricate pH-sensitive ENMs for smart beverage packaging [2], textiles [3,4], tissue engineering [5,6,7], detecting the microenvironment of various biological processes [8] and microscopic observations of microorganisms [9]. Schueren et al. [10] used polyspinning to develop pH-sensitive polyamide 6.6 nanofibers for a limited range of pH values. Agarwal et al. [11] produced nylon ENMs containing five different pH indicators in specific proportions to cover a wide range of pH values. These smart ENMs detect changes in solution pH (from pH 1 to 10) based on characteristic colors for each pH. Schuern et al. [12] developed pH-sensitive polycaprolactone (PCL) and PCL/chitosan nanofibrous structures, noting that inclusion of 20% chitosan reduced pH response time from more than 3 h to around 5 min. Rostami et al. [4] also reported successful fabrication of polyamide (PA) 6 and PCL ENMs equipped with pH sensitive dyes that detect variations in solution pH. Although they demonstrated that fibrous structure influences halochromic properties, the same sensing behavior was reported for both PA and PCL. These membranes are only useful only for detecting pH changes in a solution medium; however, detecting pH changes in vapor and at low concentrations of agent molecules is also important. 

In addition, previous works have used hydrophobic polymers with a uniform nanofiber diameter for the production of sensor nanofibers, and some reports allude to the potential of super-hydrophilic polymers in hydrogel form for use in pH-sensitive applications [13,14]. Mounting evidence also indicates that fiber membranes with a bimodal diameter distribution are more efficient at trapping water vapor molecules [15] and aerosol particles [16]. Bimodal fiber diameter ENMs from hydrophilic-hydrophobic polymers have great potential for increasing the pH sensitivity of the resulting membranes.

In the present study, polyurethane (PU) and poly (2-acryloylamido-2-methylpropanesulphonic acid) (PAMPS) were used as polymers with hydrophobic and hydrophilic properties, respectively. PAMPS is a water-soluble and ionic polymer with a high degree of hydrophilicity, and is often touted as a superabsorbent material for PC [17,18] and wound dressing [19,20,21] applications. The sulfonate group of PAMPS gives the polymer a degree of hydrophobicity and an anionic quality. The hydrophilic nature of PAMPS leads to a final structure with enhanced water absorption and transport properties. Fine nanofibers in the range below 100 nm have been electrospun from PAMPS because of its electrolytic nature [20,21]. Despite the good water absorption and fine diameter of PAMPS nanofiber membranes, the mechanical and dimensional stability of these structures is not sufficient to warrant their use in end products such as pH-sensitive membranes. This problem can be solved by blending PAMPS with PU. PU is a popular polymer that is used extensively in electrospinning processes [22,23,24]. This polymer has unique properties such as excellent hydrolytic stability, good mechanical properties, and resistance to chemicals, microorganisms and abrasion [25,26,27]. PU has hydrophobic properties and lends well to nanofibrous structures with diameters ranging from 200 to 600 nm. The effect of graphene oxide (GO) on the sensing behavior of final structures has also been investigated. Due to its excellent mechanical, thermal, and electrical properties, and the presence of hydrophilic groups (e.g., hydroxyl, carbonyl) on the surface, GO can improve the water vapor permeability and adsorption of water of nanocomposites [17,18]. Hua and coworkers showed that polyvinyl alcohol (PVA) combined with GO exhibited higher pH sensitivity, and in hydrogel form can be used for loading and selectively releasing drugs at physiological pH [19].

Against this backdrop, the present study aims to fabricate and characterize bimodal fiber nanofibrous membranes by electrospinning one hydrophobic polymer (PU) and one hydrophilic polymer (PAMPS). GO was also used to improve the response time of nanofibrous membranes with respect to detecting pH. The membranes were fabricated by simultaneous spinning of two different polymers from two opposing nozzle setups. The diameter distribution was measured by scanning electron microscopy (SEM). The pH-sensing behavior of the bimodal PU/PAMPS/GO nanofibrous membranes was investigated in both solution and vapor tests.

## 2. Materials and Methods

### 2.1. Materials

All chemicals were purchased from commercial sources and used as received unless noted otherwise. N,N-dimethylformamide (DMF), tetrahydrofuran (THF), and 2-acrylamido-2-methyl-1-propanesulfonic acid (AMPS, monomer, ≥99%) were obtained from Daejong Co. (Shiheung, South Korea). 2,2-azobisisobutyronitrile (AIBN) and four pH-indicator dyes (bromophenol blue, bromocresol green, methyl red, bromophenol red) were supplied by Sigma-Aldrich (St. Louis, MO, USA). PU was obtained from Byer (Leverkusen, Germany). Graphite was obtained from Daejong Co. (Shiheung, South Korea). Other materials including concentrated sulfuric acid, hydrochloric acid, hydrogen peroxide, sodium nitrate and potassium permanganate (KMnO_4_) were purchased from Merck (Darmstadt, Germany).

### 2.2. Preparation of Electrospinning Solutions

The four pH-indicator dyes were selected to cover a range in pH values, indicated by color changes (Table 1). Two different polymer solutions were prepared: PU solution containing the four dyes and PAMPS solution containing GO nanolayers. For the PU solution, the dye mixture (PU/dye = 99:1 w/w %) was dispersed in 5 mL of THF/DMF (60:40) with 1 h ultrasonication. PU was dissolved in THF/DMF at 30 °C for 24 h, and each dye was then added to the PU/DMF/THF solution to give an overall 6 wt% PU in THF/DMF. GO nanosheets were prepared according to the modified Hummer method. Briefly, graphite powder (2 g) and NaNO_3_ (1 g) were added into H_2_SO_4_ (46 mL) and stirred in a three-neck flask placed in an ice bath for 2 h. Then, KMnO_4_ (6 g) was added gradually under stirring and the temperature of the mixture was kept below 20 °C. The solution was heated to 35 °C and kept for 2 h. After that, distilled water (92 mL) was slowly added and the temperature was kept below 100 °C. After 15 min, an adequate amount of distilled water and 5 mL of 30% H_2_O_2_ solution were added to wash the residual KMnO_4_. Finally, the mixture was filtered and washed with 5% HCl aqueous solution and water until the pH value of the upper layer suspension reached 7. Furthermore, sonication with a cylindrical tip was used for exfoliation of graphite oxide to GO. The sonication process was carried out in room temperature (25 °C) for 20 min. Detailed information with respect to GO and PAMPS/GO formation is reported in our previous study [28]. 

### 2.3. Electrospinning

**Figure 1:**
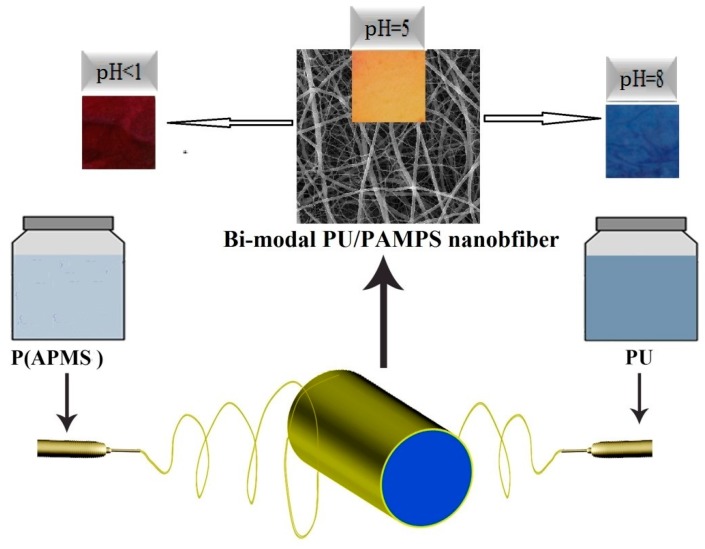
Schematic of the electrospinning process.

The simultaneous electrospinning of different precursor solutions from two syringes produced PU and PAMPS nanofibers. For this two-nozzle electrospinning, two syringes, two pumps and two high-voltage power supplies were used. One syringe contained 5 mL of PU solution containing dye while the other syringe contained 5 mL of PAMPS/GO solution. Each needle (22G; L = 34 mm and D = 0.4 mm) was connected to the positive electrode of a high-voltage power supply. The distance between the needle tips and the collector was 14 cm for each nozzle. For the first nozzle, PU solution was pumped at a rate of 0.5 mL/h and the voltage was set at 12 kV. For the second nozzle, PAMPS/GO solution was pumped at a rate of 0.1 mL/h and the voltage was set at 14 kV. Samples with different PAPMS/PU/GO ratios were obtained by changing the electrospinning time of each polymer according to Table 2. A take-up speed of 32 m/min was used for the collection of electrospun nanofibers. The electrospinning process was carried out at room temperature (22 ± 2 °C) and relative humidity (40% ± 5%). The electrospinning process is shown schematically in Figure 1.

### 2.4. Membrane Characterization

**Figure 2:**
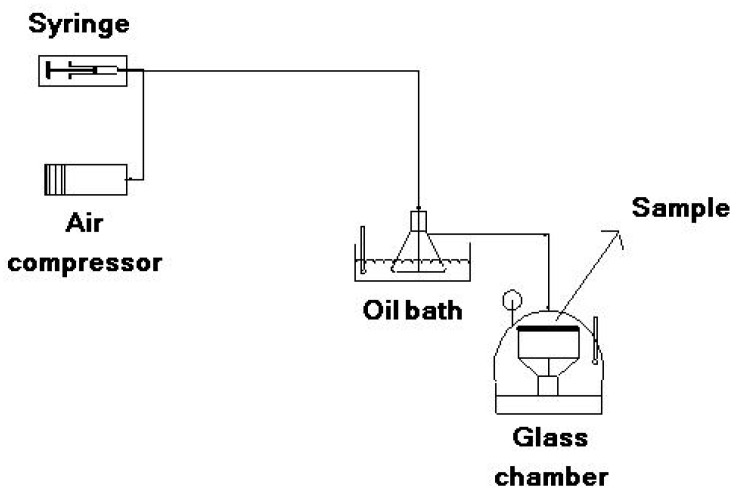
Test instrument for vapor sensing.

To obtain the colors of the dye mixture at each pH, 0.01 g of dye was dissolved in 10 mL of DMF with 1 h ultrasonication. A total of 1 mL of the prepared solution was added to a set of buffer solutions with different pH values (pH = 1, 2, 3, 4, 5, 6, 7 and 8). To determine halochromic behavior of the electrospun membranes, the solution tests were performed by dipping the membranes in each of these same buffer solutions (pH 1–8) for 2 s. Vapor tests were conducted using a homemade apparatus at three pH values (Figure 2). The desired buffer (pH = 1, 4, 8) was injected by a syringe pump and diluted by air flow provided by an air compressor (20 L/min). The injection rate was regulated to achieve a final flow with a concentration of 1000 ppm. The buffer flow was heated by an oil bath to a temperature of 90 °C and evaporated. The evaporated buffer was then conducted to a glass chamber and put in contact with the sample until a color change was detected. Solution and vapor tests were repeated three times for each sample.

The morphology of the electrospun nanofibers was characterized using transmission electron microscopy (TEM, Zeiss-EM10C Zeiss, Zaventem, Belgium) with an accelerating voltage of 80 kV, and field emission scanning electron microscopy (FE-SEM; Mira3-XMU, TESCAN, Kohoutovice, Czech Republic). The mean fiber diameter and diameter distribution were measured from SEM images using an image-processing program (ImageJ, Bethesda, Maryland, USA).

Images of reference dye solutions and electrospun nanofibers for each pH were obtained with a digital camera (Canon EOS 400D, Brampton, ON, Canada) under the identical lighting environment and camera setting. The RGB and CIELAB values (International Commission of Illumination (CIE) color space coordinates) were measured using Adobe^®^ Photoshop^®^ CS5 (San Jose, CA, USA) by taking the average of 100 pixels from 10 points in each image. The total color difference (Δ*E*) was calculated to compare the samples in different pH buffer solutions. Δ*E* was determined using according to:

(1) $$\Delta E=\sqrt{{\left(\Delta L\right)}^{2}+{\left(\Delta a\right)}^{2}+{\left(\Delta b\right)}^{2}},$$

where Δ*L* represents the brightness difference between samples, Δ*a* is the redness difference between samples, and Δ*b* is the yellowness difference between samples [2,11]. The UV-Vis spectra of all samples were recorded in a wavelength range from 380 to 780 nm with a spectrophotometer (Perkin-Elmer, Waltham, MA, USA; Model Lamba 950 or Model Lamba 45).

## 3. Results and Discussion

### 3.1. Morphology of Nanofibers

#### 3.1.1. Effect of Hydrophilic Moieties (PAMPS) on Fiber Morphology

**Figure 3:**
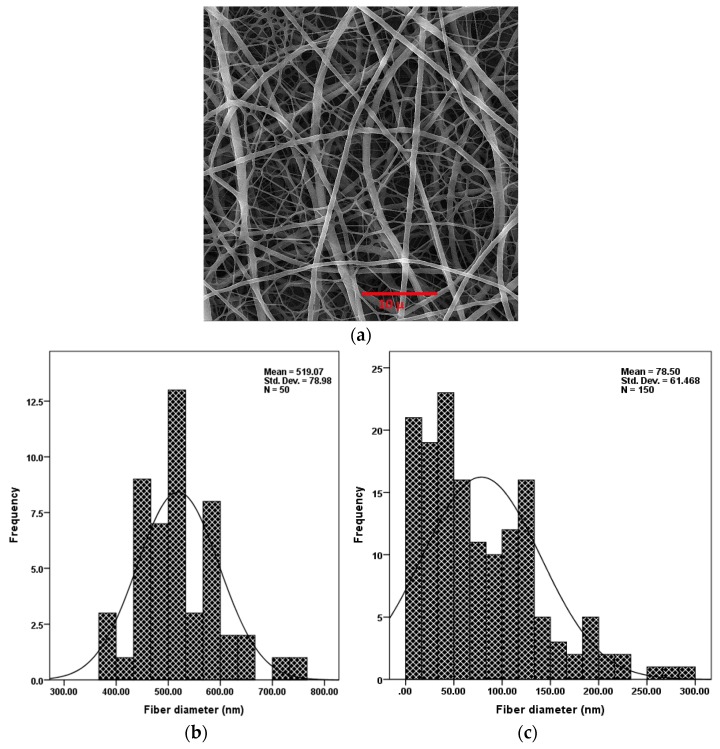
Scanning electron microscopy (SEM) image of PU/PAMPS hybrid nanofibers (**a**) and diameter distribution of PU (**b**) and PAMPS (**c**) nanofibers.

Parameters of the electrospinning process (i.e., the concentration of polymer in solution, applied voltage, nozzle to collector distance) were optimized to create non-uniform nanofibers without any structural defects such as beads. Figure 3 (top) is a SEM image showing the distribution of electrospun PU/PAMPS hybrid nanofibrous membranes. Based on SEM images, the mean diameter of the PU and PAMPS fibers was determined to be 519 and 78 nm, respectively. This difference is due in part to the fact that PAMPS is an anionic polymer containing sulfonic acid, the electrical charge of which causes an increase in charge density in the electrospinning solution. As the charge within the electrospinning jet increases, higher elongation forces are imposed on the jet under the electrical field, resulting in thinner fibers [29].

The graphs at the bottom of Figure 3 show the electrospinning process created fibers with a bimodal diameter distribution. This distribution of fiber diameter is highly favoured in the PC sector [15], for filtration [16,30], and for creating mats/meshes for tissue engineering [26] and cellular infiltration [31] applications. As the fiber diameter decreases, so does the pore diameter [30]. This is particularly useful in some cases, such as PCs, where improved windproof properties of membranes are highly desired, or in wound dressing, where warding off aerosol particles and viruses is important. Furthermore, as the diameter of the nanofibers decreases, the specific surface area increases [27]; this, in turn, improves the sensing performance of nanofibrous membranes. 

#### 3.1.2. Effect of GO on Morphology

**Figure 4:**
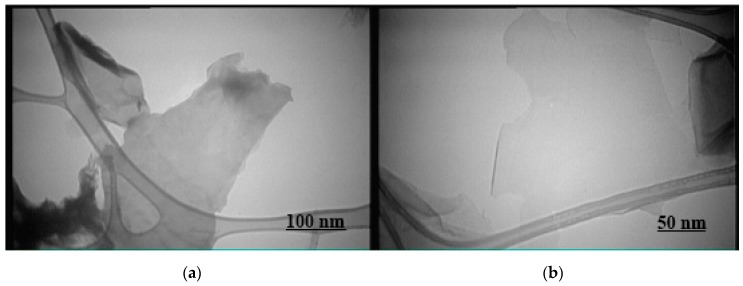
Transmission electron microscopy (TEM) image of graphene oxide: 80,000× (**a**), 100,000× (**b**).

Images were generated at different magnifications using transmission electron microscopy (TEM) to evaluate the nanoparticles synthesized with GO (Figure 4). In these images, more light passes through the nanoparticles (i.e., the particles are more transparent) when the distance between the layers of graphene oxide is greater. The graphene nanoparticles are synthesized in very thin layers, so greater transparency indicates proper synthesis of these particles.

**Figure 5:**
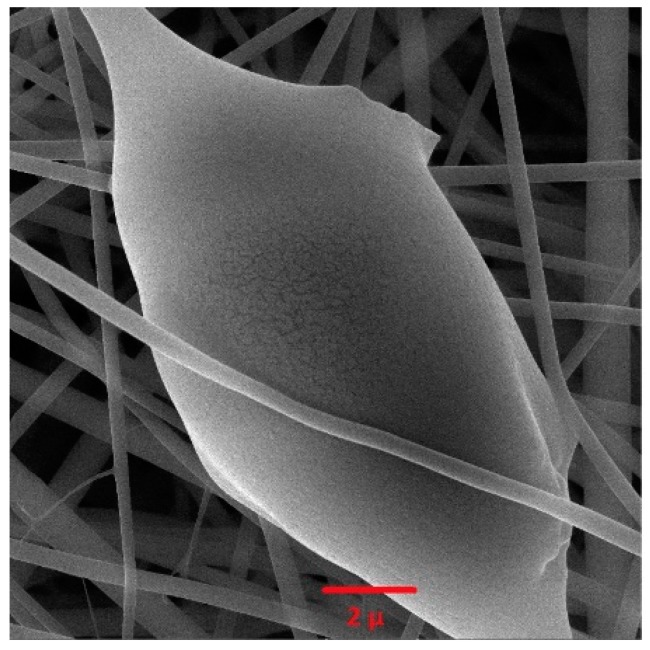
SEM image of PAMPS nanofiber containing graphene oxide.

Figure 5 shows SEM image of electrospun nanofibrous membranes containing GO (sample 40/4). The selected sample image shows that the GO is well placed within the fiber structure, with a uniform distribution. The non-uniformity in nanofibrous membranes due to the presence of graphene oxide, i.e., the areas having a notably increased diameter, is well defined in the image.

### 3.2. Halochromic Behavior of ENMs with Different Polymer Ratios

#### 3.2.1. Effect of the Hydrophilic Moiety (PAMPS) on Sensing Time

Sensing time is an important factor with respect to the pH sensitivity of ENMs. Table 3 shows the effect of the hydrophilic moiety (PAMPS) on sensing time in solution and vapor tests.

Table 3 shows that response time in solution decreases with increasing PAMPS concentration. This increase to ultra-fast sensing is attributed to the super-hydrophilicity of the PAMPS nanofibers. Although sensing time in the vapor also declined with increasing PAMPS concentration, the decrease is not enough to introduce ENMs for sensing pH in vapor media. As the response time of sample created from 40/60 PU/PAMPS was the fastest, it was carried forward to further tests.

#### 3.2.2. pH Sensing Behaviour of Reference Dye System and ENMs

**Figure 6:**
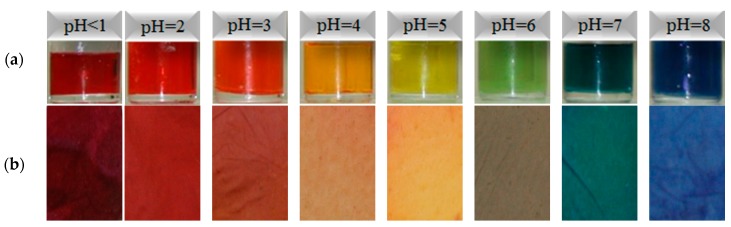
Color change in different pH buffer solutions: (**a**) reference dye solution and (**b**) samples of 40/60 PU/PAMPS.

Figure 6 shows images of the reference dye solution and ENM samples created from 40/60 PU/PAMPS in different pH buffer solutions. Minimal color difference is evident between the reference dye solution and the membrane in each pH solution. The photos show a significant color change from red to blue for the pH 1–8 interval. Notably, color development resulted after dipping the ENMs in each pH solution for only 20 s. Other studies have reported different times from 3 s to 5 min for color development [11,12]. 

**Figure 7:**
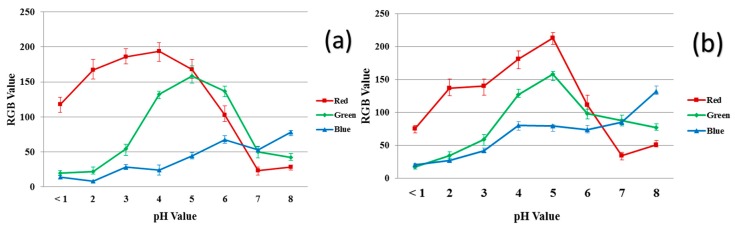
RGB values for the color produced in the different pH buffer solutions: (**a**) reference dye solution and (**b**) 40/60 PU/PAMPS.

Figure 7 shows the RGB values of 40/60 PU/PAMPS ENM samples and the reference dyes at different pH values. Increasing the acidity of the solution increases the amount of red in the samples while increasing the alkalinity increases the amount of blue. Similar trends in the curves for the 40/60 PU/PAMPS samples and the reference dye at any measured pH interval are also evident (Figure 7a vs. Figure 7b). The similarity in the trends determined for the reference dyes and the nanocomposite fibers strongly suggest the successful incorporation of all dye molecules in the ENMs [11]. 

Based on the color data (Figure 7a,b), the total color difference (Δ*E*) between different pH buffer solutions was calculated for both the reference dye solution and the electrospun nanofibers (Table 4 and Table 5). The Δ*E* values were consistently higher than 12 with the exception of the pH 2 and 3 reference dye solutions. Δ*E* values greater than 5 indicate the difference is visible to the naked eye; Δ*E* values greater than 12 indicate the two samples belongs to entirely different spaces [11]. 

**Figure 8:**
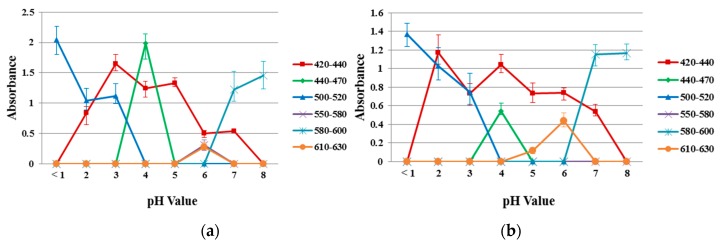
Absorption spectra in different pH buffer solutions (**a**) reference dye solution and (**b**) sample of 40/60 PU/PAMPS.

Figure 8 shows the absorption spectra of the reference dye solution and a 40/60 PU/PAMPS sample in the different pH (1–8) buffer solutions for comparison. According to the color wheel, complementary hue refers to the color observed for a solution that shows maximum absorption at a particular wavelength when continuous white light is the source [32]. The maximum absorption peak between 420 and 440 nm represents yellow, between 440 and 470 nm represents orange, between 470 and 500 nm represents red, between 500 and 520 nm represents purple, between 520 and 550 nm represents violet, between 550 and 580 nm represents violet-blue, between 580 and 620 nm represents blue, and 620 and 680 nm represents blue-green [11]. 

Absorption spectra for reference dye solutions and electrospun nanofiber samples at each pH were analyzed, with different peaks of varying intensities identified. Similar absorption peaks were noted in the range from 420 to 440 nm (pH 2–7), 440 to 470 nm (pH = 4), 500 to 520 nm (pH 1–3), 580 to 600 nm (pH 7–8) and 610 to 630 nm (pH = 6). This result indicates a high degree of incorporation and dispersion of the pH-indicator dyes in the nanofiber matrices [32].

### 3.3. Halochromic Behavior of ENMs with Different GO Ratios

#### 3.3.1. Effect of GO on Sensing Time

Table 5 shows the effect of the inclusion of nanoparticles (graphene oxide) on sensing time in solution and vapor tests.

Table 6 shows the response time in both solution and vapor tests decreases with increasing GO concentration. GO has hydrophilic groups such as OH and COOH, so an increasing concentration of GO results in increased hydrophilicity of the ENMs. The samples with higher concentrations of GO had ultra-fast sensing times in both the solution and vapor tests. As the response time of the 40/4 sample was the fastest, it was carried forward to further tests.

#### 3.3.2. pH Sensing Behavior of Reference Dye System and ENMs with Incorporated GO

**Figure 9:**
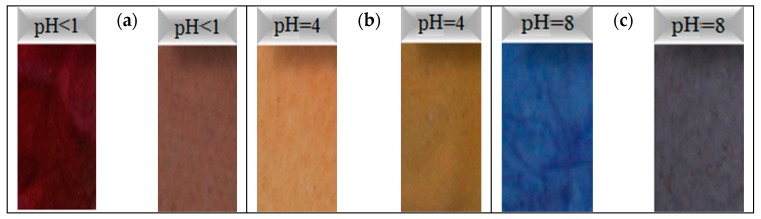
Color change in electrospun nanofibrous membrane (ENM) after solution test (left) and vapor test (right) for a sample of 40/60/4 PU/PAMPS/GO: (**a**) pH < 1, (**b**) pH = 4, (**c**) pH = 8.

Vapor tests were performed by exposing the membranes to vapor of the buffer solutions with three pH values (1, 4, 8), respectively, Figure 9 shows images of the electrospun nanofiber membranes (sample 40/4) used in the vapor test alongside those used in the solution test, and demonstrates the noticeable color difference between the three pH values considered. This color change is due to the variation of pH value, which is associated with the ionization of the carboxyl group caused by the surface charge densities of GO [19].

**Figure 10:**
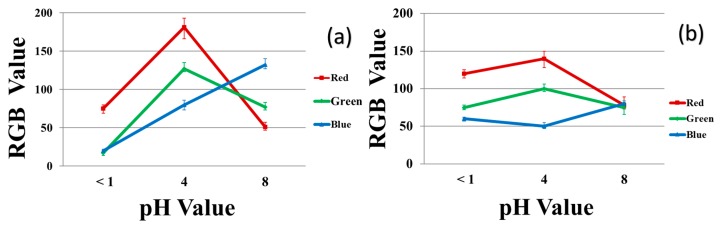
RGB values for the color produced in the different pH buffer solutions for 40/60/4 PU/PAMPS/GO samples: (**a**) solution test and (**b**) vapor test.

Figure 10 plots the RGB values for the 40/4 samples exposed to each of three pH buffer solutions in the solution (left) and vapor (right) tests. These results also show significant differences between the three pH values considered. The slight difference between the RGB values at pH 8 in the solution test vs. the vapor test is attributed to evaporation of the buffer solution.

**Figure 11:**
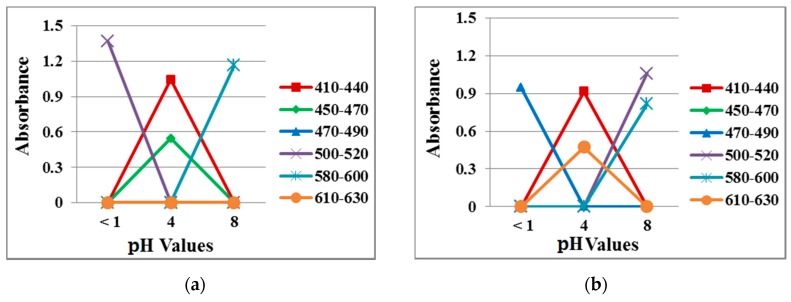
Absorption spectra of 40/4 samples exposed to different pH buffer solutions in the (**a**) solution test and (**b**) vapor test.

Figure 11 plots data from the absorption spectra measured at the three different pH values in the solution and vapor tests. At pH 1, the absorption peak at 500 nm in the solution test shifted to 480 nm in the vapor test. At pH 4, the absorption peak was noted at 620 nm in the solution test but at 460 nm in the vapor rest, suggesting more blue instead of orange in the membrane. At pH 8, the vapor test had an additional absorption peak at 500 nm, which produced a violet color. It is noted that in the vapor state, the pH value became different due to the interference of the open-air atmosphere, leading to the different penetrability properties in the membrane. As a result, slight changes in the absorption peak occurred. 

These test results show that PU/PAMPS/GO ENMs are able to sense pH variation in the surrounding environment. These results are in accordance with Schoolaert et al. [33], who used hydrophilic dye-functionalized chitosan and poly(ε-caprolactone) and reported the nanofibrous membranes show high and reproducible pH sensitivity in response to change in pH in aqueous medium and when exposed to acidic or basic gases. However, the polymers they used did not have appropriate mechanical properties, i.e., tensile strength and elongation, for use in the protective clothing sector. In addition to decreasing sensing time as demonstrated here, our previous study shows inclusion of GO can increase the mechanical properties of nanostructures with the results showing that the addition of 0.2% GO into membranes can increase the tensile strength by 65% [34]. In many applications, such as PC for the chemical industry, agricultural workers, and military textiles, external hazards are in the gas form and detectors with excellent sensing properties are required.

## 4. Conclusions

pH-sensitive dyes incorporated into ENMs were developed as a sensor for the PC sector. PU/PAMPS/GO nanofibers, loaded with pH-sensitive dyes, were successfully generated using two opposing electrospinning nozzle setups. A bimodal diameter distribution of nanofibers with pH-sensing ability was achieved, as was a high degree of incorporation and dispersion of pH-indicator dyes in the nanofiber matrix. PU/PAMP samples without GO incorporated demonstrated a rapid halochromic response in a pH range from 1 to 8. The hybrid ENMs fabricated with GO successfully responded to pH variation in a vapor test. The hydrophilicity of the ENMs was enhanced due to inclusion of PAMPS, which also resulted in thinner fibers, and GO was responsible for a faster and more sensitive response. These hybrid nanofibers have significant potential for practical applications in sectors related to protective clothing, wound dressing and drug delivery.

## Figures and Tables

**Table 1:** pH-indicator dye and corresponding pH range and color variation.

pH-Indicator Dye	pH Range	Color Change
Bromophenol blue	(Y) 3.0–4.6 (BV)	yellow to purple
Bromocresol green	(Y) 3.8–5.4 (B)	yellow to blue
Methyl red	(R) 4.2–6.2 (Y)	red to yellow
Bromophenol red	(Y) 5.2–6.8 (R)	yellow to red

**Table 2:** Samples with different concentrations of polyurethane (PU), poly 2-acrylamide-2-methylpropanesulfonic acid (PAMPS) and graphene oxide (GO).

Sample Code	PU/PAMPS	GO (w/w %)
100	100/0	0
80	80/20	0
60	60/40	0
40	40/60	0
40/1	40/60	1
40/2	40/60	2
40/3	40/60	3
40/4	40/60	4

**Table 3:** The effect of PAMPS on pH sensing time.

Sample Code	PU/PAMPS	Solution Response Time (s)	Vapor Response Time (s)
100	100/0	80	300
80	80/20	40	170
60	60/40	30	100
40	40/60	20	60

**Table 4:** Total color difference (ΔE) observed between different pH buffer solutions exposed to the reference dye solution.

	pH = 2	pH = 3	pH = 4	pH = 5	pH = 6	pH = 7	pH = 8
pH < 1	23.94	26.04	50.94	66.71	69.89	64.1	47.37
pH = 2		8.77	45.98	68.62	81.05	86.34	67.36
pH = 3			40.46	63.26	76.49	84.82	66.8
pH = 4				25.63	49.59	82.93	61.21
pH = 5					30.1	76.71	57.46
pH = 6						53.11	41.47
pH = 7							29.68

**Table 5:** Total color difference (ΔE) observed between different pH buffer solutions exposed to the sample of 40/60 PU/PAMPS.

	pH = 2	pH = 3	pH = 4	pH = 5	pH = 6	pH = 7	pH = 8
pH < 1	28.37	25.14	47.58	64.83	36.82	53.45	57.15
pH = 2		11.87	37.54	50.73	46.27	72.62	78.06
pH = 3			27.86	43.49	34.7	61.94	69.84
pH = 4				18.84	28.91	58.22	74.71
pH = 5					44.58	72.04	92.05
pH = 6						30.08	51.83
pH = 7							38.61

**Table 6:** Effect of GO on sensing time.

Sample Code	Solution Response Time (s)	Vapor Response Time (s)
40/1	5	15
40/2	3	12
40/3	0	9
40/4	0	7
